# Insulin resistance, age and depression’s impact on cognition in middle-aged adults from the PREVENT cohort

**DOI:** 10.1136/bmjment-2023-300665

**Published:** 2023-05-26

**Authors:** Sarah D Bauermeister, Michael Ben Yehuda, Graham Reid, Gregory Howgego, Karen Ritchie, Tam Watermeyer, Sarah Gregory, Graciela Muniz Terrera, Ivan Koychev

**Affiliations:** 1 Department of Psychiatry, Medical Sciences Division, University of Oxford, Oxford, UK; 2 INSERM, Institut des Neurosciences de Montpellier, Montpellier, France; 3 Edinburgh Dementia Prevention, The University of Edinburgh Centre for Clinical Brain Sciences, Edinburgh, UK

**Keywords:** depression & mood disorders, delirium & cognitive disorders, psychiatry

## Abstract

**Background:**

Alzheimer’s disease (AD), type 2 diabetes mellitus (characterised by insulin resistance) and depression are significant challenges facing public health. Research has demonstrated common comorbidities among these three conditions, typically focusing on two of them at a time.

**Objective:**

The goal of this study, however, was to assess the inter-relationships between the three conditions, focusing on mid-life (defined as age 40–59) risk before the emergence of dementia caused by AD.

**Methods:**

In the current study, we used cross-sectional data from 665 participants from the cohort study, PREVENT.

**Findings:**

Using structural equation modelling, we showed that (1) insulin resistance predicts executive dysfunction in older but not younger adults in mid-life, that (2) insulin resistance predicts self-reported depression in both older and younger middle-aged adults and that (3) depression predicts deficits in visuospatial memory in older but not younger adults in mid-life.

**Conclusions:**

Together, we demonstrate the inter-relations between three common non-communicable diseases in middle-aged adults.

**Clinical implications:**

We emphasise the need for combined interventions and the use of resources to help adults in mid-life to modify risk factors for cognitive impairment, such as depression and diabetes.

**WHAT IS ALREADY KNOWN ON THIS TOPIC**

- Mood disorders and metabolic diseases are known to be frequently comorbid. Furthermore, both conditions are known to be associated with cognitive impairment and cognitive decline. There has been some evidence that the risk of cognitive impairment associated with diabetes and depression is most notable in mid-life. However, studies focusing on this period of life have been sparse, and most research has modelled bivariate correlations among cognitive impairment, depression and diabetes. As such, this study was conducted in order to model the inter-relations between the three conditions in a large cohort, while focusing on mid-life as depression and diabetes in this period are thought to carry a higher risk of cognitive impairment.

**WHAT THIS STUDY ADDS**

- While insulin resistance, as a core feature of diabetes, was related to depression across all stages of mid-life, the relationship with cognitive functioning was more complex. In the current study, we found that the stage of mid-life in which middle-aged adults find themselves moderates the relationship between insulin resistance and cognition and depression and cognition; that is, only in older middle-aged adults does insulin resistance predict impaired cognition (ie, executive function) and does depression predict impaired cognition (ie, visuospatial memory).

**HOW THIS STUDY MIGHT AFFECT RESEARCH, PRACTICE OR POLICY**

- Clinicians should be mindful of the impact of comorbidities between cognitive impairment, metabolic diseases, such as diabetes, and mood disorders, such as depression in mid-life. Given the risk of intractable dementia in individuals with cognitive impairment, available resources for intervening in modifiable risk factors, such as depression and diabetes, should be considered for adults in the middle period of life.

## Introduction

The prevalence of Alzheimer’s disease (AD) and type 2 diabetes mellitus (T2DM) is reaching epidemic proportions across the globe. Indeed, numerous studies have shown that those with T2DM are at risk of developing AD and that the AD brain, in turn, becomes even poorer at processing glucose as the disease progresses.^1^ Central nervous system deficits in glucose processing are defined as central insulin resistance, which typically impacts the brain’s ability to support basic psychological functioning, including cognition and mood.^2^ However, studies have also shown that with comorbid AD, psychological dysregulations associated with brain insulin resistance occur at an enhanced degree.^3^ Likewise, healthy adults without a T2DM diagnosis, but with higher levels of insulin resistance, also have a higher risk of abnormal cognitive and affective functioning.^4^ Longitudinally, those with higher levels of insulin resistance, even in the absence of a T2DM diagnosis, have a higher risk of AD just 3 years later.^5^ These examples illustrate the relationship between insulin resistance and T2DM, whereby the former is a risk factor for the latter and can occur years before formal diagnosis of T2DM.^6^ Yet, while there is clear evidence of a link between insulin resistance and cognitive impairment, there remains a paucity of research exploring related variables, such as affective disorders, which would enhance our understanding of the relationship between AD and T2DM in ageing populations.^7^

A better understanding of dementia risk factors and their interactions is a priority, given the inefficacy of available treatments and the fact that dementia-related brain changes occur decades before the expression of any dementia symptoms.^8^ Indeed, it is estimated that up to 35% of dementia cases are attributable to preventable risk factors.^9^ Over the last 10 years, there has been increasing interest in the metabolic aspects of AD, focusing mainly on the dysregulation of glucose, as well as some lipid compounds. As for T2DM, research has suggested that a T2DM diagnosis carries a 1.5 times higher risk of non-vascular dementia compared with the general population.^10^ Indeed, patients with AD seem to have reduced peripheral insulin sensitivity and resting hyperinsulinaemia, with evidence that their cognitive function may be improved by inducing further hyperinsulinaemia while maintaining euglycaemia.^11^ This suggests a chronic alternation in patients’ metabolic state, leading to cognitive impairment with at least some degree of reversibility as demonstrated by a recent case–control study showing reduction in dementia incidence with diabetic agents that cross the blood–brain barrier.^12^ Animal models of both AD and insulin resistance have similar phenotypes in terms of brain insulin handling, receptor expression and resistance.^13^ A number of mechanisms for the effect of diabetes on cognition and dementia risk have been suggested. For example, there is some evidence that T2DM, a state of peripheral insulin resistance, is associated with increased Aβ deposition^14^; however, insulin may be acting through other mechanisms such as by increasing inflammation, oxidative stress, vascular pathology or though altered glucose and lipid metabolism, thereby increasing the likelihood of an AD diagnosis in those with T2DM.

As for related comorbidities, the presence of T2DM more than doubles the odds of comorbid depression, and depression worsens the prognosis, mortality and treatment compliance of diabetic patients.^15^ There is also a small but growing body of evidence which suggests diabetes and depression may act additively to increase the risk of dementia.^16^ For example, one study has shown that while diabetes and depression differentially impact cognitive processes, such as memory and executive function, together they significantly accelerate the general overall rate of decline, especially in individuals 50–64 years of age.^17^ With age-related decreases in cognitive functioning, any additional processing burden caused by affective symptoms, along with the effects of poor blood glucose control, may prove detrimental to cognitive processing. Dementia, depression and T2DM are thus three common non-communicable disorders which often coexist, negatively interact with each other and may share pathophysiological mechanisms. Existing evidence suggests mid-life is when preventable risk factors such as depression and poor T2DM control exert the largest effect on dementia risk.^9^ However, the exact nature of the interaction between T2DM, depression and dementia has not been directly characterised in this age group. Thus, in the current study, we used the baseline data from the PREVENT study cohort of individuals 40–59 years of age to examine the relationship between insulin resistance, cognitive function and depression in mid-life prior to a dementia diagnosis.

## Materials and methods

### Participants

A total of 665 participants were included in the current paper from the PREVENT cohort, which recruited individuals aged 40–59 from four centres (West London, Edinburgh, Cambridge and Oxford) in the period 2014–2019.^18^ The participants were all cognitively healthy at recruitment (ie, no diagnosis of cognitive impairment) as assessed by self-report and formal cognitive testing conducted via interview, as well as the Addenbrooke’s Cognitive Assessment III.

### Computerised cognitive tasks

Participants completed a neuropsychological assessment from the COGNITO battery at their baseline visit. This battery is delivered on a touchscreen device and has been designed for the detection of both normal and pathological cognitive changes from adolescence onwards with previous research demonstrating its acceptability and reliability.^19 20^ From the battery of assessments, participants undertook tasks which included measures of processing speed (reaction time), episodic memory (recall), and phonemic and semantic fluency. Visuospatial orientation was assessed through the 4 Mountains Test. Intraindividual reaction time variability measured as an intraindividual SD (ISD) measure within the speed task was included as a sensitive measure of early neurobiological change.^21^

### Insulin resistance

Insulin and glucose concentrations were determined by analysis of fasting plasma samples obtained at the first study appointment. Insulin resistance was calculated using the homeostatic model assessment for insulin resistance (HOMA-IR): fasting plasma glucose (mmol/L) times fasting serum insulin (mU/L) divided by 22.5.

### Depressive symptom burden

The level of affective symptoms was determined using the Center for Epidemiological Studies–Depression Scale (CES-D). CES-D is a 20-item measure which asks respondents to indicate the extent to which they have experienced various symptoms of depression, scoring each item from 0 (rarely/none) to 3 (most of the time). Scores range from 0 to 60 with higher scores indicating greater depressive symptom burden.

### Statistical analysis

#### Preprocessing

All data processing and statistical analyses were performed in Stata SE V.16.1. Cognitive tasks were log-transformed to normalise the distribution where appropriate. Insulin resistance was also log-transformed and two extreme outliers were trimmed for this variable (>99.9 percentile).

#### Structural equation model (SEM)

To assess the direct and indirect effects between insulin resistance, depression and cognition, a SEM was used. The aim of the SEM analysis was to assess the shared mechanistic pathways underlying insulin resistance, depression and cognition in a single model. The aim of the SEM was also to assess the prediction pathways of insulin resistance and depression on selected individual cognitive tasks and of insulin resistance on depression. Executive function was assessed as a latent construct formed from the semantic and phonemic fluency tasks, which were initially z-transformed. A measure of ISD of reaction time was computed from the processing speed task and was included in the model as a proxy measure of neurocognitive integrity.^21^ Depression was included in the model as the total CES-D score and insulin resistance was entered as the HOMA-IR value. To adjust for the confounding effects of age, education and sex, these were also included in the model as covariates.

##### Cognitive variables

A latent construct for reflecting executive functioning (language) was formed from the two indicator measures of phonemic verbal and semantic verbal fluency.^22^ Both indicators are sensitive indicators of cognitive change over time as well as early indicators of mild cognitive impairment (MCI) and dementia.^23^ Although both were correlated with each other (r=0.298, p<0.001), a covariance relationship was not required in the model (non-multicollinearity) as the level correlation did not affect model estimation. Processing speed was entered into the model as an individual task measured as the average of 12 reaction time trials (ms). The 4 Mountains Test score was entered into the model as a measure of spatial memory, and a delayed recall of names score was entered into the model as an assessment of delayed recall over time. An ISD metric of the reaction time task was computed to reflect a proxy measure of neurocognitive integrity. Of note is that it is also a sensitive indicator of cognitive change over time, as well as an early indicator of MCI and dementia.^24^

##### Covariate variables

To adjust for the confounding effects of age, education and sex, these were entered into the SEM model as covariates. Direct paths were extended between the covariates and the cognitive variables. Age was included into the model as a continuous variable. Based on hypothesised stronger effect in older adults, we subsequently divided age by decade, that is, 40–49 vs 50–59 for the group analyses (maintaining age as continuous variable to account for the effect of age within groups). Sex was coded such that women were 0 and men were 1, and education was included as a continuous variable (number of years).

##### Predictor variables

Insulin resistance was included in the model as a value in milligram per minute, and depression was included as participants’ total CES-D score.

##### Estimation and fit

The model was estimated using the maximum likelihood estimation method with missing values (mlmv) method with standardised beta coefficients. The mlmv method assumes joint normality and that missing values are missing at random.

## Results

The participant sample had a mean age of 51.20 years (SD=5.44), and 61.65% of the participants were female. Baseline participant characteristics are presented in table 1.

**Table 1:** Participant characteristics

	**N**	**Mean (SD)**
Demographics		
Age	665	51.20 (5.44)
Sex (% female)	665	61.65
Education (years)	661	16.67 (3.39)
Cognition		
4 Mountains Test	423	10.41 (2.34)
Processing speed	645	5756.29 (1516.21)
Phonemic fluency	645	11.32 (4.12)
Semantic fluency	644	16.40 (4.15)
Recall	644	6.90 (1.47)
ISD	554	0.14 (0.07)
Affective		
CES-D	659	9.26 (8.46)
Biomarkers		
Insulin resistance	644	60.02 (40.85)
Lifestyle factors		
Body mass index	656	27.75 (5.60)
Hours spent sleeping	656	6.74 (1.05)
Anti-inflammatory drugs (%)	656	0.03
Parental dementia (%)	656	51.5

CES-D, Center for Epidemiological Studies–Depression Scale; ISD, intraindividual SD.

### Pairwise correlations

A pairwise correlation with Bonferroni correction was conducted and showed few significant associations between the cognitive variables of interest (outcome variables) and predictor/mediator variables (insulin resistance and depression). The full correlation table output is presented in online supplemental materials S1 but to be noted are the significant associations between insulin resistance and semantic verbal fluency (r=−0.18, p<0.01), between insulin resistance and sex (r=0.16, p<0.01) where females were coded as zero and between insulin resistance and depression (r=0.15, p<0.01).

10.1136/bmjment-2023-300665.supp1 Supplementary data

### Structural equation model

#### SEM measurement regression paths

**Figure 1:**
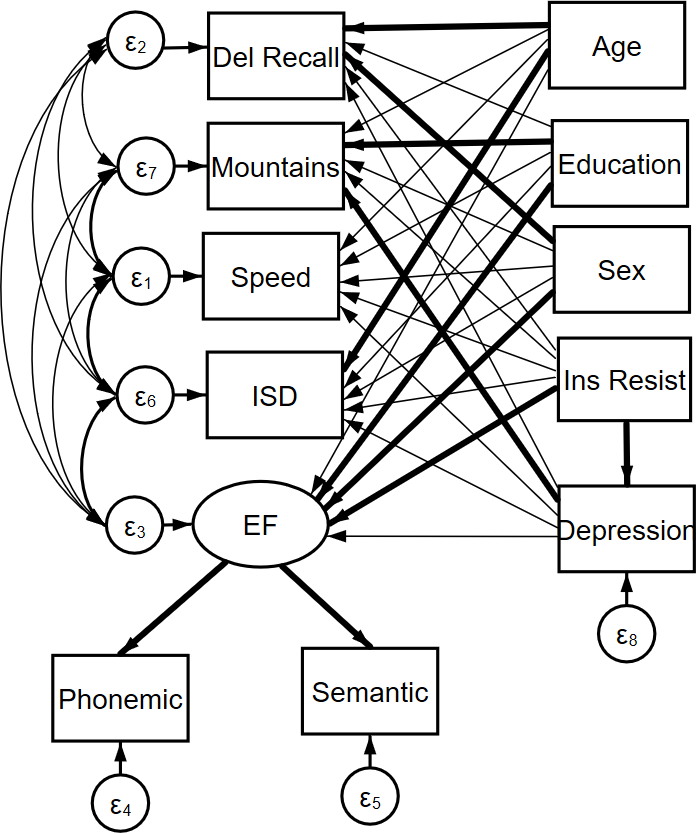
Structural equation model path diagram. The latent construct for EF in our analyses is constructed from language-focused variables. EF, executive function; ISD, intraindividual SD.

A direct path was extended from insulin resistance to each of the individual cognitive variables and the executive function latent construct. A direct path was also extended from depression to each of the cognitive variables and to the executive function latent construct to assess the relationship between depression and these variables. To assess mediation by depression on the relationship between insulin resistance and cognition, a direct path was also inserted between insulin resistance and depression (figure 1).

#### SEM results

**Figure 2:**
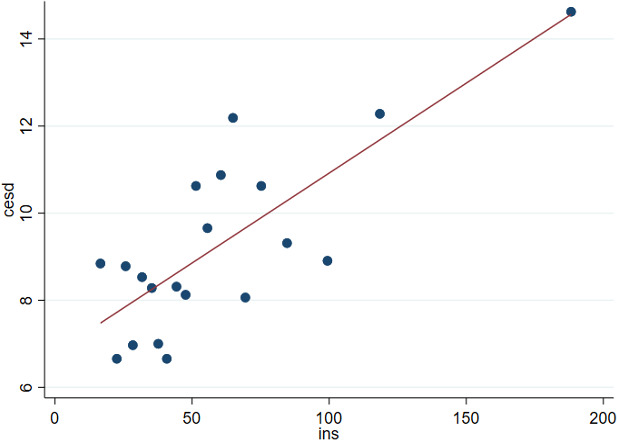
Visualisation of the linear relationship between depression and insulin resistance using a binned scatter plot. CES-D refers to depression scores. CES-D, Center for Epidemiological Studies–Depression Scale; INS, insulin resistance as measured by homeostatic model assessment for insulin resistance.

The full SEM output is presented in online supplemental materials S2, where the beta values are presented as standardised values. The results showed that higher insulin resistance values significantly predicted lower executive function performance (b=−0.12, p<0.01), and higher insulin resistance predicted increased depressive scores (b=0.15, p<0.001) (see figure 2). Insulin resistance was not associated with performance in any other cognitive tasks or ISD, but increased depressive scores predicted poorer performance on the 4 Mountains Test (b=0.14, p<0.01). The model was repeated by age group (age 40–49 vs age 50–60); for the older age group, the relationship between lower insulin resistance and executive function remained significant (b=−0.15, p<0.01), but for the younger age group, this relationship was non-significant (b=−0.09, p=0.126). For both age groups, higher insulin resistance predicted increased depressive scores (p<0.01), whereas higher depressive scores predicted poorer performance in the 4 Mountains Test (p<0.05) for only the older age group.

10.1136/bmjment-2023-300665.supp2 Supplementary data

Goodness-of-fit measures for the initial full group model were deemed good by field standards: root mean squared error of approximation (RMSEA)=0.02, Comparative Fit Index (CFI)=0.983, Tucker Lewis Index (TLI)=0.925, χ^2^=15.80, p=0.148. RMSEA is a metric of the differences between the predicted outcomes of the model and the observed values in the data. CFI is a metric of the improvement in a model’s fit going from the baseline model to the proposed model, which is less sensitive to differences in sample sizes. TLI is a measure of the relative reduction in misfit per additional degree of freedom in the model.

## Discussion

The aim of the current study was to explore the inter-relationships between cognitive functioning, depression and insulin resistance in cognitively healthy middle-aged adults. Using structural equation modelling, we found that lower insulin resistance values predicted higher executive function performance while controlling for the effects of age, education and sex. We also found that higher insulin resistance values predicted increased self-reported depressive scores. When we re-estimated our models across different age groups (here, those aged 40–49 years and 50–60 years), we found that the significant relationship between insulin resistance and executive function was apparent for the older age group but not for the younger age group, suggesting that higher insulin resistance in older middle-aged adults associates with aspects of cognition, including executive function. As for the age-related effects on the relationship between insulin resistance and depressive symptoms, our results revealed that higher levels of insulin resistance were associated with higher levels of depressive symptoms in both age groups. Moreover, finally, we found that higher levels of depressive symptoms were linked to lower scores of visuospatial navigation skills (or spatial memory) in the older group but not in the younger group of middle-aged adults.

Concerning the negative association between insulin resistance and executive function, our results corroborate a growing body of literature, suggesting that those with higher levels of insulin resistance have greater executive dysfunction.^25^ When considered against the lack of such effects on tasks probing episodic memory, the current results suggests that the link is primarily through insulin sensitivity associating with a preservation of cognitive reserve broadly, possibly through cerebrovascular factors, rather than a relationship with a specific neurodegeneration aetiology (eg, AD). This interpretation is supported by data linking T2DM to risk for vascular rather than AD dementia.^26^ In our analysis, we observed that age is a factor in the relationship between insulin resistance and cognition, which disagrees with the findings of other papers.^27^ Given that executive functions are not a unified and homogenous set of neuropsychological constructs, it is possible that the moderating effect of age on the relationship between insulin resistance and executive function holds only for some subtypes of executive functioning but not for others, such as shifting and inhibition.^27^ Second, while we used a continuous measure of insulin resistance as a possible indicator of diabetes risk, others have used a categorical measure of diabetic status in which only 41 participants of the 465 included in their sample had T2DM.^27^ Such use of T2DM caseness as a proxy of insulin resistance is likely underpowered to detect relationships between insulin resistance and cognitive outcomes.

Turning now to our findings of a relationship between greater levels of insulin resistance and higher scores of self-reported depressive symptoms, in both younger and older adults, the results of the current study fit within the state of the current literature.^28–30^ Theorists have proposed several pathways to understand the link between depression and insensitivity to insulin. In recent years, depression has been further characterised as a (partly) inflammatory disorder owning to the potential role of psychosocial and environmental stress in triggering depressive episodes. In response to significant stressors, the body produces higher levels of cortisol that, although inflammatory, are thought to support acute fight or flight responses.^31^ With chronic stress and persistently elevated levels of stress hormones in the bloodstream, there can be metabolic dysfunction of carbohydrates in the body.^32^ Thus, stress-induced hypercortisolaemia can result in elevated levels of glucose, which is a major pathway in the development of T2DM. Factors known to associate with both insulin resistance and depression, such as sleep quality, as well as hormones (eg, oestrogen), may also interact with this relationship.^6^

Several limitations of the currently reported findings exist. First, while the observed association of executive dysfunction with insulin resistance points to likely cerebrovascular pathophysiology, the study lacks biomarker data relevant to dementia to clarify the nature of any potential neurodegenerative processes. Second, while we chose a well-validated measure of insulin resistance in HOMA-IR, the gold standard remains the hyperinsulinaemic euglycaemic clamp (HEC). It is therefore possible that the reliability of the insulin resistance scores could have been improved by the use of a HEC. The third methodological caveat is that it is now recognised that there is a distinction between central and peripheral insulin resistance, which has relevance to human behaviour. HOMA-IR as well as HEC only allow assessment of peripheral insulin resistance; it is possible that the link between depressive scores, insulin resistance and cognition could be clarified further through methods of assessing central insulin resistance specifically. Finally, a number of factors are known to play a role in both depression and insulin resistance (eg, obesity, sleep and oestrogen). We have not been able to explore the mediating link of these factors through lack of relevant data availability but should be part of future research in the area.

## Conclusions

Given the evident comorbidities, the current study sought to further explore the inter-relationships between cognitive function, depressive symptoms, and insulin resistance. Using data from over 600 participants from the PREVENT prospective cohort study, our analyses revealed that insulin resistance is associatesd with executive dysfunction in older but not younger middle-aged adults, in addition to depression scores in both age groups. As for cognitive functioning, depression could predict visuospatial navigation abilities, as measured by athe 4 Mountains taskTest, in the older but not younger middle-aged adult group. Together, we have shown connections between three common diseases that place an emotional, health, and socioeconomic burden on individuals and on society at large. Further research using prospective cohorts such as the one reported here couldcan inform on the longitudinal relationships between these factors, thus clarifyinginforming the potential for multi-domain interventions targeting specific at-risk groups.

## Data Availability

Data are available upon reasonable request. Data may be obtained from a third party and are not publicly available. Data are available in the prospective PREVENT cohort.

## References

[1] Arnold SE , Arvanitakis Z , Macauley-Rambach SL , et al . Brain insulin resistance in type 2 diabetes and Alzheimer disease: concepts and conundrums. Nat Rev Neurol 2018;14:168–81. 10.1038/nrneurol.2017.185 29377010 PMC6098968

[2] Lee S-H , Zabolotny JM , Huang H , et al . Insulin in the nervous system and the mind: functions in metabolism, memory, and mood. Mol Metab 2016;5:589–601. 10.1016/j.molmet.2016.06.011 27656397 PMC5021669

[3] Talbot K . Brain insulin resistance in Alzheimer’s disease and its potential treatment with GLP-1 analogs. Neurodegener Dis Manag 2014;4:31–40. 10.2217/nmt.13.73 24640977 PMC4465775

[4] Reijmer YD , van den Berg E , Ruis C , et al . Cognitive dysfunction in patients with type 2 diabetes. Diabetes Metab Res Rev 2010;26:507–19. 10.1002/dmrr.1112 20799243

[5] Schrijvers EMC , Witteman JCM , Sijbrands EJG , et al . Insulin metabolism and the risk of Alzheimer disease: the Rotterdam study. Neurology 2010;75:1982–7. 10.1212/WNL.0b013e3181ffe4f6 21115952 PMC3014236

[6] Ley SH , Schulze MB , Hivert MF , et al . Risk factors for type 2 diabetes. In: Diabetes in America. 2018.

[7] Bauermeister S , Bunce D . Poorer mental health is associated with cognitive deficits in old age. Neuropsychol Dev Cogn B Aging Neuropsychol Cogn 2015;22:95–105. 10.1080/13825585.2014.893554 24605782

[8] Ojakäär T , Koychev I . Secondary prevention of dementia: combining risk factors and scalable screening technology. Front Neurol 2021;12:772836. 10.3389/fneur.2021.772836 34867762 PMC8634660

[9] Livingston G , Huntley J , Sommerlad A , et al . Dementia prevention, intervention, and care: 2020 report of the Lancet Commission. Lancet 2020;396:413–46. 10.1016/S0140-6736(20)30367-6 32738937 PMC7392084

[10] Chatterjee S , Peters SAE , Woodward M , et al . Type 2 diabetes as a risk factor for dementia in women compared with men: a pooled analysis of 2.3 million people comprising more than 100,000 cases of dementia. Diabetes Care 2016;39:300–7. 10.2337/dc15-1588 26681727 PMC4722942

[11] Craft S , Newcomer J , Kanne S , et al . Memory improvement following induced hyperinsulinemia in Alzheimer’s disease. Neurobiol Aging 1996;17:123–30. 10.1016/0197-4580(95)02002-0 8786794

[12] Wium-Andersen IK , Osler M , Jørgensen MB , et al . Antidiabetic medication and risk of dementia in patients with type 2 diabetes: a nested case-control study. Eur J Endocrinol 2019;181:499–507. 10.1530/EJE-19-0259 31437816

[13] Akter K , Lanza EA , Martin SA , et al . Diabetes mellitus and Alzheimer’s disease: shared pathology and treatment? Br J Clin Pharmacol 2011;71:365–76. 10.1111/j.1365-2125.2010.03830.x 21284695 PMC3045545

[14] Stanciu GD , Bild V , Ababei DC , et al . Link between diabetes and Alzheimer’s disease due to the shared amyloid aggregation and deposition involving both neurodegenerative changes and neurovascular damages. J Clin Med 2020;9:1713. 10.3390/jcm9061713 32503113 PMC7357086

[15] Bădescu SV , Tătaru C , Kobylinska L , et al . The association between diabetes mellitus and depression. J Med Life 2016;9:120–5. 27453739 PMC4863499

[16] Sullivan MD , Katon WJ , Lovato LC , et al . Association of depression with accelerated cognitive decline among patients with type 2 diabetes in the ACCORD-MIND trial. JAMA Psychiatry 2013;70:1041–7. 10.1001/jamapsychiatry.2013.1965 23945905 PMC4212406

[17] Demakakos P , Muniz-Terrera G , Nouwen A . Type 2 diabetes, depressive symptoms and trajectories of cognitive decline in a national sample of community-dwellers: a prospective cohort study. PLoS ONE 2017;12:e0175827. 10.1371/journal.pone.0175827 28414754 PMC5393617

[18] Ritchie CW , Ritchie K . The prevent study: a prospective cohort study to identify mid-life biomarkers of late-onset Alzheimer’s disease. BMJ Open 2012;2:e001893. 10.1136/bmjopen-2012-001893 PMC3533047 23166135

[19] de Roquefeuil Guilhem RK . COGNITO: computerized assessment of information processing. J Psychol Psychother 2014;04:1–7. 10.4172/2161-0487.1000136

[20] Kahali B , Balakrishnan A , Dhanavanthri Muralidhara S , et al . COGNITO (computerized assessment of adult information processing): normative scores for a rural Indian population from the SANSCOG study. Alzheimers Dement 2022:1–10. 10.1002/alz.12572 36516088

[21] Bunce D , Bauermeister S . Oxford research encyclopedia of psychology. Intraindividual reaction time variability, attention, and age-related outcomes. Oxford University Press, 2019.

[22] Amunts J , Camilleri JA , Eickhoff SB , et al . Comprehensive verbal fluency features predict executive function performance. Sci Rep 2021;11:6929. 10.1038/s41598-021-85981-1 33767208 PMC7994566

[23] Frankenberg C , Weiner J , Knebel M , et al . Verbal fluency in normal aging and cognitive decline: results of a longitudinal study. Computer Speech & Language 2021;68:101195. 10.1016/j.csl.2021.101195

[24] Haynes BI , Bauermeister S , Bunce D . A systematic review of longitudinal associations between reaction time Intraindividual variability and age-related cognitive decline or impairment, dementia, and mortality. J Int Neuropsychol Soc 2017;23:431–45. 10.1017/S1355617717000236 28462758

[25] Crochiere RJ , Lansing AH , Carracher A , et al . Executive function and somatic problems in adolescents with above target glycemic control. Pediatr Diabetes 2019;20:119–26. 10.1111/pedi.12789 30345593 PMC6331243

[26] Doney ASF , Bonney W , Jefferson E , et al . Investigating the relationship between type 2 diabetes and dementia using electronic medical records in the godarts bioresource. Diabetes Care 2019;42:1973–80. 10.2337/dc19-0380 31391202

[27] Yeung SE , Fischer AL , Dixon RA . Exploring effects of type 2 diabetes on cognitive functioning in older adults. Neuropsychology 2009;23:1–9. 10.1037/a0013849 19210028 PMC2712627

[28] Anderson RJ , Freedland KE , Clouse RE , et al . The prevalence of comorbid depression in adults with diabetes: a meta-analysis. Diabetes Care 2001;24:1069–78. 10.2337/diacare.24.6.1069 11375373

[29] Katon W , Von Korff M , Ciechanowski P , et al . Behavioral and clinical factors associated with depression among individuals with diabetes. Diabetes Care 2004;27:914–20. 10.2337/diacare.27.4.914 15047648

[30] Golden SH , Lazo M , Carnethon M , et al . Examining a bidirectional association between depressive symptoms and diabetes. JAMA 2008;299:2751–9. 10.1001/jama.299.23.2751 18560002 PMC2648841

[31] Bozovic D , Racic M , Ivkovic N . Salivary cortisol levels as a biological marker of stress reaction. Med Arch 2013;67:374–7. 10.5455/medarh.2013.67.374-377 24601177

[32] Katsu Y , Baker ME . Cortisol. In: Handbook of hormones. Academic Press, 2021: 947–9.

