# Supplementary material for: Insulin resistance, age and depression’s impact on cognition in middle-aged adults from the PREVENT cohort
Source: BMJ Ment Health. 2023 May 26;26(1):e300665. doi: 10.1136/bmjment-2023-300665 (PMC10231438; doi:10.1136/bmjment-2023-300665)
Supplement: Supplementary data [file bmjment-2023-300665supp001.pdf]

Supplementary Material 1

Bivariate Correlation Table

	Age	Sex	EDUC	IR	CESD	DR	MOUNT	RT	VF PH	VF SEM
Age										
Sex	.06									
EDUC	-.11	-.08								
IR	.12	.16*	-.10							
CESD	-.004	-.02	-.04	.15*						
DR	.11	.31*	-.09	.07	.07					
MOUNT	.03	-.02	-.11	.05	.15	.10				
RT	.06	-.02	-.03	.05	.02	-.02	.04			
VF PH	-.06	-.06	.11	-.04	-.05	-.17*	-.06	.006		

BMJ Publishing Group Limited (BMJ) disclaims all liability and responsibility arising from any reliance placed on this supplemental material which has been supplied by the author(s)

VF SEM	-.05	-.33*	.22*	-.19*	-.05	-.22*	-.10	-.05	.30*	

Abbreviations: EDUC (Education in years); IR (Insulin Resistance); CESD (Epidemiological Studies-Depression Scale); MOUNT (4 Mountains Task); RT (Reaction Time); VF PH (Phonemic Verbal Fluency); VF SEM (Sematic Verbal Fluency)
