# Supplementary material for: Insulin resistance, age and depression’s impact on cognition in middle-aged adults from the PREVENT cohort
Source: BMJ Ment Health. 2023 May 26;26(1):e300665. doi: 10.1136/bmjment-2023-300665 (PMC10231438; doi:10.1136/bmjment-2023-300665)
Supplement: Supplementary data [file bmjment-2023-300665supp002.pdf]

Supplementary Material 2

SEM Output

	Processing Speed				
	B	SE	z	CI	
CES-D score	.02	.04	.38	-.06	.09
Insulin resistance	.05	.04	1.28	-.03	.13
Education	-.02	.04	-.48	-.10	.06
Age	.06	.04	1.49	-.02	.14
Sex	-.04	.04	-.90	-.11	.04
	Delayed Recall				
CESD score	.07	.04	1.92	.00	.15
Insulin resistance	.00	.04	.09	-.07	.08
Education	-.05	.04	-1.36	-.13	.02
Age	.09*	.04	2.40	.02	.16
Sex	.30***	.04	8.29	.23	.37
	4 Mountains Task				

CESD score	.14**	.05	3.06	.05	.23
Insulin resistance	.03	.05	.53	-.07	.12
Education	-.12*	.05	-2.52	-.21	-.03
Age	.02	.05	.50	-.07	.12
Sex	-.05	.05	-.97	-.14	.05
	**Executive Function**				
CESD score	-.03	.04	-.82	-.11	.05
Insulin resistance	-.12**	.04	-2.95	-.20	-.04
Education	.19***	.05	4.07	.10	.28
Age	-.01	.04	-.36	-.09	.06
Sex	-.31***	.05	-6.20	-.41	-.21
	**Within-person Variability**				
CESD score	.02	.04	.35	-.07	.10
Insulin resistance	.05	.05	1.12	-.04	.14

Education	-.04	.04	-.96	-.13	.04
Age	.12**	.04	2.75	.03	.20
Sex	-.04	.04	-.85	-.12	.05
	**Insulin Resistance**				
CES-D score	.15***	.04	4.03	.08	.23
